# Temperature Alters Host Genotype-Specific Susceptibility to Chytrid Infection

**DOI:** 10.1371/journal.pone.0071737

**Published:** 2013-08-26

**Authors:** Alena S. Gsell, Lisette N. de Senerpont Domis, Ellen van Donk, Bas W. Ibelings

**Affiliations:** 1 Department of Aquatic Ecology, Netherlands Institute of Ecology (NIOO-KNAW), Wageningen, The Netherlands; 2 Department of Biology, University of Utrecht, Utrecht, The Netherlands; 3 Microbial Ecology, Institut F.-A. Forel, Université de Genève, Versoix, Switzerland; Leiden University, Netherlands

## Abstract

The cost of parasitism often depends on environmental conditions and host identity. Therefore, variation in the biotic and abiotic environment can have repercussions on both, species-level host-parasite interaction patterns but also on host genotype-specific susceptibility to disease. We exposed seven genetically different but concurrent strains of the diatom *Asterionella formosa* to one genotype of its naturally co-occurring chytrid parasite *Zygorhizidium planktonicum* across five environmentally relevant temperatures. We found that the thermal tolerance range of the tested parasite genotype was narrower than that of its host, providing the host with a “cold” and “hot” thermal refuge of very low or no infection. Susceptibility to disease was host genotype-specific and varied with temperature level so that no genotype was most or least resistant across all temperatures. This suggests a role of thermal variation in the maintenance of diversity in disease related traits in this phytoplankton host. The duration and intensity of chytrid parasite pressure on host populations is likely to be affected by the projected changes in temperature patterns due to climate warming both through altering temperature dependent disease susceptibility of the host and, potentially, through en- or disabling thermal host refugia. This, in turn may affect the selective strength of the parasite on the genetic architecture of the host population.

## Introduction

Parasitism is one of the most common consumer strategies [1] and can impose large fitness costs on host individuals and populations. However, the level of host susceptibility to disease often depends on the biotic and abiotic environmental context [2]. This interdependency between host, parasite and their shared environment was first formulated in the disease triangle concept [3]. Environmental conditions affect the population dynamics of hosts and parasites, but also the strength and nature of the host-parasite interaction [4]. Variation in the environmental context such as nutrient enrichment, can, for example, shift the character of the interaction from mutualism to antagonism in plants and their mycorrhizal fungi [5]. Environmental variability can also cause more subtle changes in the strength of host-parasite interactions by slowing down or disrupting parasite mediated directional selection on the host population [4]. Moreover, environmental variability can also maintain genetic diversity in disease related traits of the host if the disease resistance of a host genotype varies with environmental conditions so that no genotype is overall the most or least susceptible to disease across all environments [6]. In that case, no host genotype can out-compete all others permanently as the fitness based ranking order of genotypes varies across environmental gradients in space and/or time [7].

Temperature is probably the most pervasive environmental variable and influences the metabolic rates of all organisms [8], [9]. Nevertheless, the specific temperature effects on host-parasite interactions are diverse. Depending on parasite physiology, lower temperatures can increase parasite infectivity [10], decrease disease severity [11] or halt infection altogether [12]. The relationship between temperature and parasite infectivity is of specific interest in fungal diseases which have been recognized an emerging infectious disease threat [13]. Changing environmental temperature patterns are thought to influence the infectivity and spread of several fungal diseases in animal and plant hosts, among which also important food crops [14]. The fungal phylum Chytridiomycota (commonly referred to as chytrids) has gained notoriety as the chytrid *Batrachochytriumdendrobatidis* is the causative agent of amphibian chytridiomycosis, one of the main drivers of worldwide population declines in amphibians [15]. Chytrids are cosmopolitan and occur in a wide range of habitats and substrates, acting as saprophytes but also as parasites (and even hyper-parasites) on hosts as diverse as bacteria, phytoplankton, vascular plants, invertebrates and vertebrates [16]–[20]. While the chytrid species parasitizing amphibians seems to be a generalist, most chytrid species parasitizing phytoplankton hosts are highly host specific [21], [22]. Although chytrid occurrence and biomass is probably underreported [23], a few host-chytrid systems are relatively well described, in particular the spring-bloom diatom *Asterionella formosa* Hassall and its two chytrid parasites: *Zygorhizidium planktonicum* Canter and *Rhizophydium planktonicum* Canter emend [24]–[26]. *Asterionella* often is a prominent contributor to the diatom spring bloom in lakes worldwide. Its blooms are frequently followed by chytrid epidemics with prevalence of infection exceeding 90% in many cases [12], [25]. Field observations showed that the development of *Asterionella* spring-blooms depends on water temperatures in early spring as *Asterionella* already reproduces at temperatures below 3°C, while the parasite is still inactive [26]. This mismatch in thermal ranges provides the host with a low temperature window of disease-free population growth which bears consequences for the size the diatom spring-bloom [12] and its genetic structure [27]. Warmer winters in which water temperature stays above 3°C remove this window of opportunity since the parasite remains active, denying the host the ability to build up a bloom [12]. Knowledge on variation in thermal tolerance of a wider set of chytrid parasites will help assessing the implications of climate change on host-chytrid interactions in general. We add to this by contributing a case-study on thermal reaction norms in a chytrid-diatom model-system.

Species- and genotype-level host-parasite interaction patterns are expected to depend on their environmental conditions, in particular on their temperature environment. In order to test thermal reaction norms of *Asterionella formosa* susceptibility to chytrid infection, we performed an infection experiment using seven concurrent genotypes of the diatom host exposed to a single genotype of its chytrid parasite *Zygorhizidium planktonicum* across a range of environmentally relevant temperatures. We assessed host and parasite thermal tolerance range and optima for activereproduction. Furthermore, we tested for host genotype (G) and temperature (T) main effects and for host genotype-by-temperature (GxT) interactions in net production of host and parasite. As the host genotypes also showed temperature-dependent differences in cell-size, we checked the extent of co-linearity of host cell size and genotype effects on host susceptibility to disease.

## Materials and Methods

### Host-parasite system

*Asterionella formosa* is a pennate diatom that forms uniclonal, stellate colonies. It is a characteristic spring-bloom diatom of temperate lakes [28] but can also bloom in late summer. In Lake Maarsseveen (The Netherlands, E 05° 05′ 08", N 52° 08′ 34"), *Asterionella* blooms are often followed by chytrid epidemics exceeding 90% prevalence of infection [29]. Despite predominantly (or exclusively) asexual reproduction of *Asterionella* [30], the population in Lake Maarsseveen is genetically highly diverse [27], [31]. This diversity is also reflected in phenotypic variation in fitness traits across a temperature gradient [32] and in resistance to parasitism [31].

The chytrid parasite *Zygorhizidium planktonicum* is an obligate and highly virulent parasite of the diatoms *Asterionella formosa* and *Synedraacus* Kützing [24]. Chytrid epidemics can bring *Asterionella* spring blooms to a swift end and can therefore affect the phytoplankton succession in lakes [25], [26]. Each infection prohibits host reproduction and quickly kills the host [33]. Transmission occurs by motile zoospores that actively search for host cells, guided by chemotaxis to photosynthetic exudates of their host [34]. After attachment, the zoospores grow into epibiontic sporangia, within which the next generation of zoospores is formed and eventually released by rupture of the sporangium wall [35]. Sporangia development time, zoospore production per sporangium, and zoospore infective lifetime all depend on their current temperature environment [36]. After sexual reproduction and at temperatures below 3°C, *Zygorhizidium* forms thick-walled resting spores which are inactive and allow the parasite to weather adverse periods [26].

### Isolation of experimental strains

All *Asterionella formosa* genotypes used in the experiment were isolated from a single water sample taken during the 2008 *Asterionella* spring-bloom at 5 m depth in Lake Maarsseveen. Host culture establishment was fairly unbiased with a larger than 95% success rate. As all cells of an *Asterionella* colony are the asexual offspring of a founding cell, isolating single colonies is an easy way to obtain uniclonal cultures of this diatom. Individual isolates were grown in batch culture on CHU-10 medium [37] modified with 2-fold concentrations of PO_4_ and FeCl_3_. For genetic fingerprinting, 50mL of dense culture were centrifuged, and the DNA of the remaining pellet was extracted by a modified QiagenDNeasy Plant Mini Kit (QiagenN.V.,Venlo, the Netherlands) protocol (see file S1 for DNA extraction details). Genetic fingerprinting was done by amplified fragment length polymorphism (AFLP), using four primer combinations: (i) Eco + GA &Mse + AT, (ii) Eco + GA &Mse + CC, (iii) Eco + GA &Mse + CG, and (iv) Eco + GC &Mse + AC. The AFLP fingerprinting of the seven experimental and the parasite baiting genotypes of *Asterionella formosa* as well as of one *Fragilariacrotonensis* genotype (functioning as out-group) was performed by Keygene® (Keygene N.V., Wageningen, The Netherlands), details of the AFLP data analysis are presented in the file S1 accompanying this paper.

Isolation of uniclonal *Zygorhizidium planktonicum* cultures from the same bloom/epidemic occurred by transfer of infected *Asterionella* colonies carrying only one single sporangium into a uniclonal culture of *Asterionella* S122 (Lake Maarsseveen, isolated 2008). Establishing parasite cultures was less successful with only 20 infected cultures out of over 400 isolation attempts. This lower success may suggest, that, by baiting the parasite with a uniclonal host culture, we actually screened for parasite genotypes able to infect this specific host genotype. Hence, our collection of parasite isolates may well represent a limited range of the genetic variation present in Lake Maarsseveen. Since we used only one uniclonal isolate of the parasite, we did not assess the genetic diversity of our isolate collection. Host and parasite cultures were maintained in semi-continuous batch cultures in environment test chambers (SANYO Electric, Moriguchi, Japan) at 18°C±1°C and 14: 10 h light:dark cycle at 50 µmol quanta s^–1^ m^–2^ provided by cool-white fluorescent lamps (TL-D 30W/830, Philips, Amsterdam, The Netherlands). All cultures were uniclonal but had slight bacterial contaminations.

### Experimental design and methodology

The experiment employed a full-factorial design with seven *Asterionella* genotypes (S24, S26, S37, S38, S43, S49 and S53) exposed to one *Zygorhizidium* genotype (F12) at five different temperatures (1°C, 6°C, 11°C, 16°C and 21°C±0.5°C ) in five replicates. This resulted in 35 experimental combinations and 175 experimental units. To compare the performance of *Asterionella* in parasite exposed and non-exposed populations, non-exposed controls of the seven *Asterionella* genotypes were grown at the same experimental temperatures resulting in 35 control units (one per temperature-host strain combination).

For temperature acclimation, parasite exposed and non-exposed stock cultures of each host genotype were split into five subcultures and stepwise acclimated in semi-continuous batch cultures in temperature-controlled water baths for at least five generations prior to the experiment [38]. The light was set to 160±10 µmol quanta m^–2^ s^–1^ provided by cool-white fluorescent lamps (TL-D 30W/830) at a 14: 10 light: dark cycle. Photosynthesis-by-irradiance curves (PHYTO-PAM, Heinz Walz, Effeltrich, Germany) showed that this irradiance level was saturating but not inhibiting.

To start the experiment, each experimental and control unit was inoculated from the corresponding non-exposed subculture to a starting concentration of ca. 15 000 uninfected host cells mL^–1^ into a total volume of 60 mL CHU-10 medium [37]. Parasite exposure was achieved by inoculating ca. 5 000 live infection carrying host cells mL^–1^ from a nearly 100% infected, temperature- and host genotype-matching exposed subculture. Based on results of pre-experiment trials, the host inoculum of ca 15 000 cells mL^–1^ was small enough to ensure that the culture medium could support several generations of unlimited host growth before light or nutrient availability could become limiting in the batch set up, but also large enough to support the infection. Similarly, the parasite inoculum of ca 5 000 live infection carrying cells mL^–1^ (i.e. a starting prevalence of ca 25%) was large enough to follow both increase and decrease in prevalence over time. All experimental and control units were started on the same day. Each unit was shaken manually twice and their position within the water bath was randomized once each day. Samples for microscopy enumeration were taken every second day for the temperatures 6°C to 21°C and every fifth day for temperature 1°C. All samples were taken at the same moment in the light cycle, fixed with a Glutaraldehyde-Formaldehyde mixture (to a final concentration of 0.01%) and stored cool and dark.

### Counting protocol

A minimum of 200 *Asterionella* cells or 20 fields of view were counted in a 1 mL sample under an inverted microscope (Leica, DMI 4000B, Wetzlar, Germany) according to the Utermöhl settling method [39]. Each sample was counted for abundance of: (i) living uninfected host cells mL^–1^ (*uninf*); (ii) infected host cells carrying one or more living infection(s) mL^–1^ (*inf*); (iii) infected host cells carrying only dehisced / dead infection(s) mL^–1^; (iv) sporangia mL^–1^, and (v) resting spores mL^–1^. Infection prevalence, i.e. proportion of cells carrying live infections in the live host population, was calculated as *inf/(uninf+inf).Asterionella* cells carrying empty or dead sporangia, i.e. class (iii), were excluded from the calculation of infection prevalence as they did not contribute further to population growth of the host or the parasite.

### Statistical analysis

To assess the thermal ranges of hosts and parasites, the rate of change day^–1^ for *uninf, inf*, the combined *uninf+inf* and for parasite sporangia in the experimental units, were averaged over all host genotypes and plotted against temperature. The host genotype-specific rate of change day^–1^ of each variable was calculated as
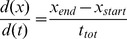
whereby *x* is the abundance of either uninfected or infected host cells and parasite sporangia on the last day of the experiment (*x_end_*) and the first day of the experiment (*x_start_*) respectively. Furthermore, *t_tot_* is the total number of experimental days.

To work with comparable experimental times and include at least three samples, the first ten days for temperature treatments 1°C to 16°C were used in the statistical analysis. For treatment 21°C only the first six days were included as the infection had cleared and the host population was approaching carrying capacity, hence analysing a longer interval would have resulted in underestimating host production. The response variables were the net production of uninfected (*P uninf*) and infected (*P inf*) host cells mL^–1^, net production of parasite sporangia mL^–1^ (*P spor*), net increase/decrease in infection prevalence (*P prev*) and the infection related percentaged reduction in production of *uninf* cells (*% reduction*). The net production (*P x*) variables were calculated as
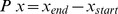



And the *% reduction* of the production of uninfected cells was calculated as
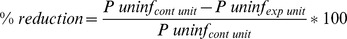



whereby *P uninf_cont unit_* is the net production of uninfected cells mL^–1^ in control units, and *P uninf_exp unit_* is the net production of uninfected cells mL^–1^ in experimental units.

Within our wide experimental temperature spectrum, all response variables showed non-linear relationships with the explanatory variable temperature. To allow for these non-linear relationships a generalised additive model (GAM) [40] was employed, using package “mgcv” [41] in R v.2.13.1 [42]. This additive model fits a smoothing curve through the data, in this case based on thin plate regression splines. As overfitting can be a problem in GAM models [40], we selected the most parsimonious model based on F-tests between models of increasing complexity, starting with the simplest model, including only the predictor “temperature treatment”. In addition, the generalised cross-validation (GCV) scores (estimating the optimal amount of smoother) were compared: the lower the GCV score of a model, the better the model fit. All variables were checked for normality and heteroscedasticity of variance prior to analyses. The variable *P inf* was sqrt-transformed to remove heteroscedasticity; however its variances did not co-vary with temperature (data not shown). All statistical analyses and plot graphing were carried out in R [42] and SigmaPlot 11.0 (Systat Software, San Jose, U.S.).

## Results

### AFLP fingerprinting

**Figure 1:**
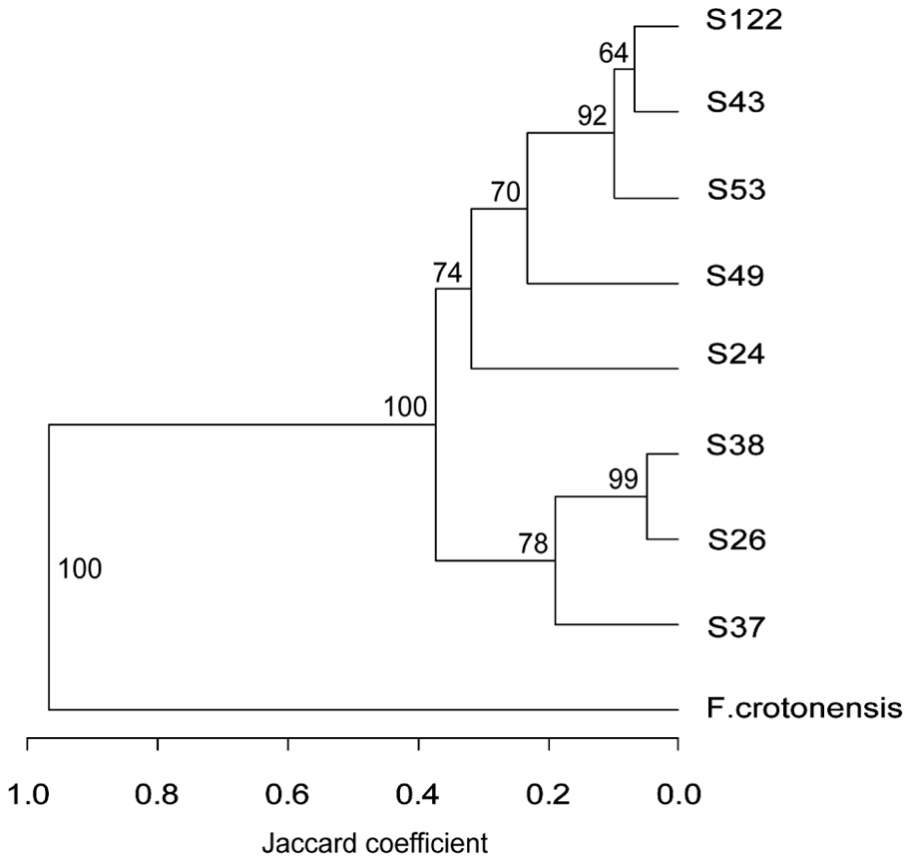
Representation of the genetic diversity of the experimental *Asterionellaformosa* genotypes. The dendrogram representation is based on Jaccard similarity among the *Asterionellaformosa* genotypes used for baiting the parasite (S122) and in the experiment (S24–S53), as well as one *Fragilariacrotonensis* genotype as out-group. Bootstrap resampling of the data (n = 5000) showed support of most of the nodes, the distinction between S122 and S43 was not supported well.

The AFLP analysis of uniclonal cultures of eight *Asterionella formosa* and one *Fragilariacrotonensis* yielded 113 marker bands, of which 69% were polymorph. Each uniclonal culture showed a unique fingerprint pattern and therefore represented a unique genotype. The UPGMA dendrogram (Fig. 1) showed *Fragilariacrotonensis* as a clear out-group and clustered the *Asterionella formosa* genotypes in broadly two clusters of three and five genotypes. The dendrogram was a good representation of the Jaccard's similarity matrix as the cophenetic correlation coefficient was r = 0.98 (Mantel test *P* = 0.001). Most of the nodes were supported well as shown by bootstrap resampling (n = 5000) results (Fig. 1). However, the distinction between S122 and S43 was not well supported. Nevertheless, given the empirical data, the presented dendrogram is the best possible representation of the data.

### Thermal tolerance ranges

**Figure 2:**
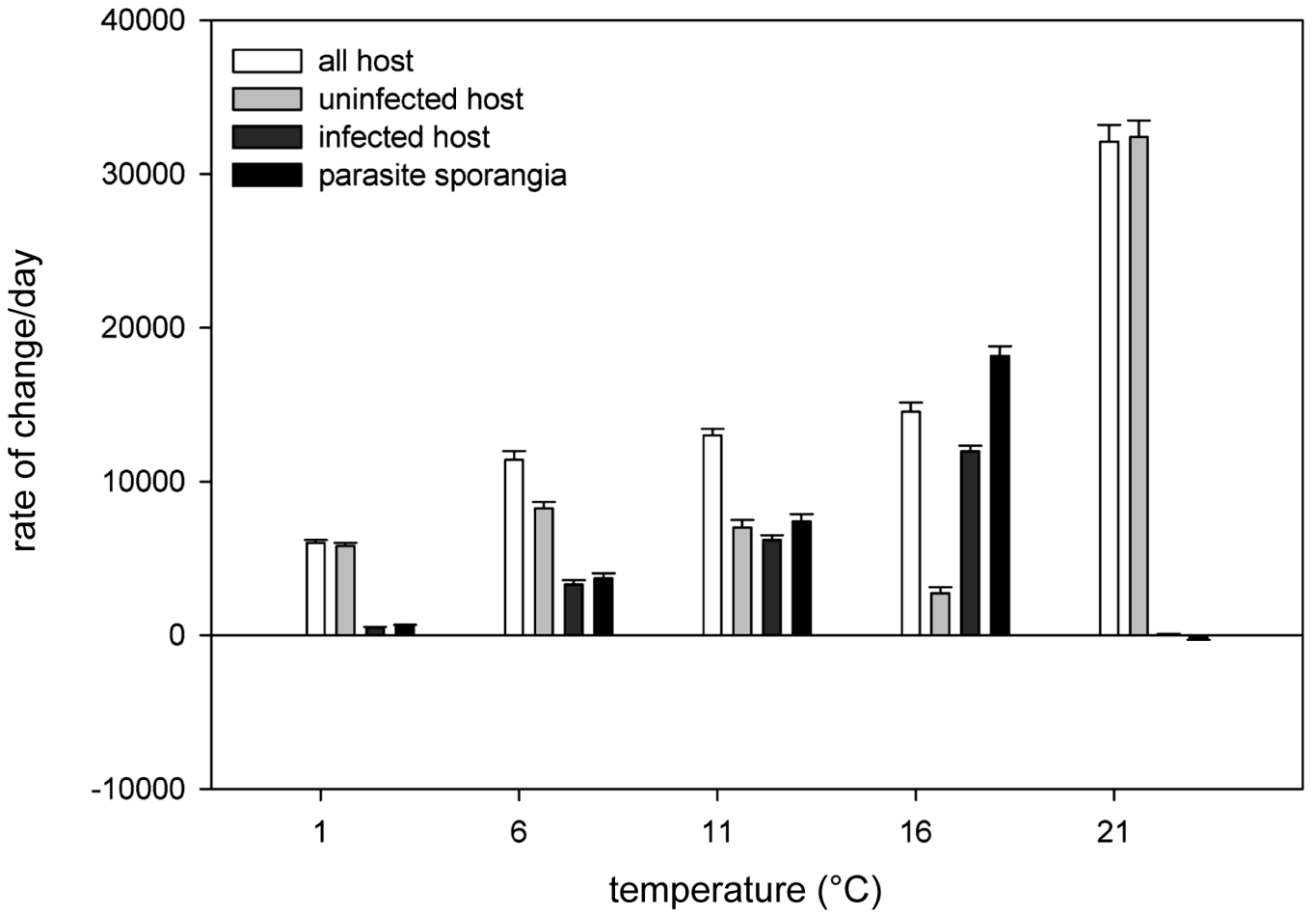
Thermal tolerance ranges of aggregated, species-level measures of host and parasite productivity across temperature environments. This plot shows overall thermal reaction norms of exposed, but uninfected (light grey) and exposed, infected (dark grey) host (expressed as a rate of change day^–1^ in *Asterionella* cells) separately and combined (no colour), as well as the thermal reaction norm of the parasite (as rate of change day^–1^ of chytrid sporangia, black bars) in experimental units. The thermal tolerance range of the tested parasite genotype is narrower than that of the host as the parasite population shows low or no growth at both temperature extremes while the host population is still productive.

To show the general temperature effect on host and parasite productivity, the rate of change per day in host abundance, (infected, uninfected and total (*uninf+inf*) in experimental units) and parasite sporangia abundance (in experimental units) were averaged across host genotypes and plotted against temperature treatment (Fig. 2). Total host and parasite productivity showed a typical left skewed, unimodal relationship across temperature with the maximal performance temperature near the upper tolerance limit. Optimum performance temperature of the host in the experiment was achieved at 21°C, that of the parasite around 16°C. The tested parasite genotype showed a narrower tolerance range than the host, and the relationship between host and parasite changed with temperature level. At the two lowest temperatures, both uninfected host and parasite showed positive net production, but the uninfected host outperformed the parasite. At temperature 1°C, parasite production occurred mainly as resting spores, which stay inactive as long as the temperature remains too low for parasite reproduction. At intermediate temperatures (11 and 16°C), the parasite outperformed the uninfected host performance, which was also reflected in large increases in infection prevalence in these treatments. But at the highest temperature the uninfected host outperformed the parasite again as the lethal temperature limit of the tested parasite genotype was surpassed. Hence, in our experiment, the host had two thermal refugia (a “cold” and a “hot” one) of very low or no parasite pressure.

### Main and interactive effects of host genotype

**Figure 3:**
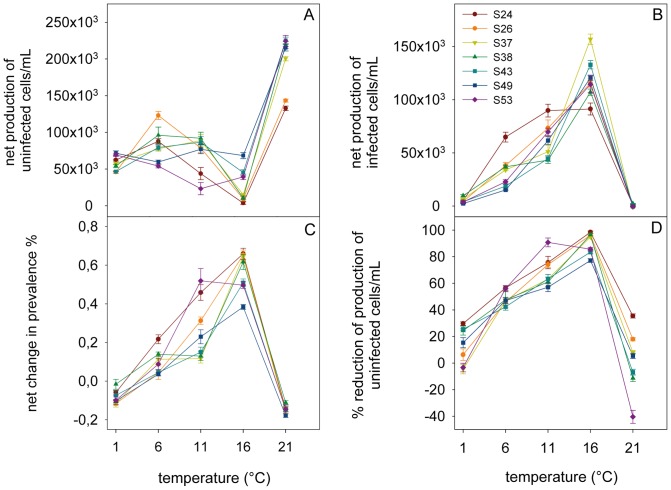
Genotype specific thermal reaction norms. Observed net production of A) exposed, but uninfected host cells mL^–1^, B) exposed, infected host cells mL^–1^, C) net change in prevalence of infection, and D) % reduction of the production of uninfected cells mL^–1^ in parasite exposed cultures, plotted as host genotype-specific thermal reaction norms. Note the changes in host genotype performance ranking order across temperatures. Such changes indicate the potential for genotype-by-temperature interactions.

**Figure 4:**
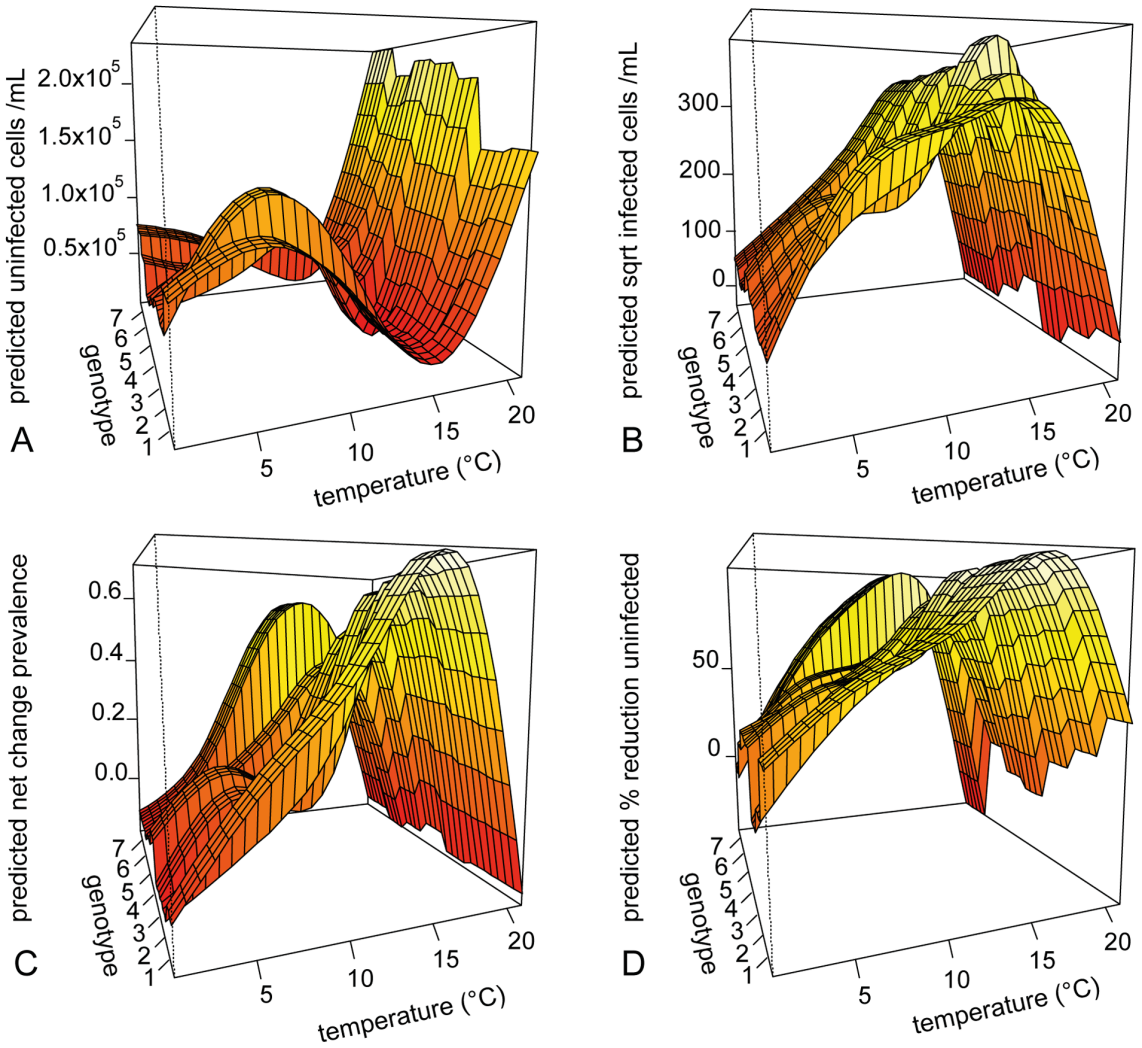
Visualisation of the GAM predictions for the measured response variables. The plots show production of A) exposed, but uninfected cells mL^–1^, B) sqrt transformed exposed, infected cells mL^–1^, C) of net change in prevalence of infection, and D) % reduction of the production of uninfected cells mL^–1^ in parasite exposed cultures.

The most parsimonious GAM model included main effects of temperature (*T*) and host genotype (*G*), as well as genotype-by-temperature interaction (*GxT*) effects (Table S1):
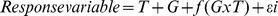



whereby we employed a non-parametric smoothing function *f* (based on thin plate regression splines), a Gaussian error distribution *εi*, and a link function by identity. Main and interaction effects were significant for each of the four response variables: net production of uninfected (*P uninf*; Table 1a) and infected (*P inf*; Table 1b) host cells mL^–1^, net increase/decrease in infection prevalence (*P prev*; Table 1c) and the infection related percentage reduction in production of *uninf* cells *(% reduction*; Table 1d). For all four response variables, the model fits were good with R^2^_adj_ between 0.936 and 0.981 (Table 1a–d). The observed thermal reaction norms of genotype-specific host response variables were visualised in Figures 3A–D, which also show the changes in genotype performance ranking order changes across temperature. Model predictions were visualised for each of the response variables as a thermal reaction norm per genotype (Fig. 4A–D).

**Table 1:** Results of the GAM for parametric effects (temperature (*T*) and genotypes a-g (*G*) and smoothed interaction (genotype by temperature (*f(GxT)*)) on net production of uninfected and infected host cells mL^–1^ in parasite exposed cultures, on net change in infection prevalence and on the net reduction of production of uninfected host cells mL^–1^.

*a) P uninf = (T) + (G) + f(GxT) + ε*				
Parametric coefficients:	Estimate	Std. Error	t value	Pr(>|t|)
(Intercept)	–155909	4557	–34.217	**<2e-16**
T	20177	389	51.852	**<2e-16**
Ga	14067	3164	4.446	**1.77e-05**
Gb	21896	3164	6.920	**1.48e-10**
Gc	28388	3164	8.972	**1.69e-15**
Gd	30577	3164	9.664	**<2e-16**
Ge	32340	3164	10.221	**<2e-16**
Gf	16088	3164	5.084	**1.16e-06**
**Approximate significance of smooth terms ** ***f(GxT)*** **:**	**edf**	**Ref.df**	**F**	**P**
s(T):Ga	3.76	3.88	227.9	**<2e-16**
s(T):Gb	3.82	3.89	212.9	**<2e-16**
s(T):Gc	3.87	3.89	184.8	**<2e-16**
s(T):Gd	3.87	3.89	185.4	**<2e-16**
s(T):Ge	3.85	3.89	144.8	**<2e-16**
s(T):Gf	3.82	3.89	155.1	**<2e-16**
s(T):Gg	3.72	3.88	193.0	**<2e-16**
GCV score = 3.0419e+08	n = 175	**R^2^_adj_ = 0.94**		

*b) P inf = (T) + (G) + f(GxT) + ε*				
Parametric coefficients:	Estimate	Std. Error	t value	Pr(>|t|)
(Intercept)	411.2	7.10	57.922	**<2e-16**
T	–20.1	0.61	–33.084	**<2e-16**
Gb	–15.6	4.92	–3.170	**0.002**
Gc	–18.0	4.92	–3.666	**0.0003**
Gd	–16.3	4.92	–3.308	**0.001**
Ge	–32.5	4.92	–6.609	**7.50e-10**
Gf	–40.0	4.92	–8.129	**2.06e-13**
Gg	–30.0	4.92	–6.105	**9.55e-09**
**Approximate significance of smooth terms ** ***f(GxT)*** **:**	**Edf**	**Ref.df**	**F**	***P***
s(T):Ga	3.86	3.89	458.7	**<2e-16**
s(T):Gb	3.88	3.89	481.1	**<2e-16**
s(T):Gc	3.89	3.89	522.0	**<2e-16**
s(T):Gd	3.88	3.89	357.9	**<2e-16**
s(T):Ge	3.88	3.89	467.6	**<2e-16**
s(T):Gf	3.87	3.89	509.4	**<2e-16**
s(T):Gg	3.87	3.89	484.1	**<2e-16**
GCV score = 378	n = 175	**R^2^_adj_ = 0.98**		

*c) P prev = (T) + (G) + f(GxT) + ε*				
Parametric coefficients:	Estimate	Std. Error	t value	Pr(>|t|)
Intercept	–0.799	0.022	36.049	**<2e-16**
T	–0.052	0.002	–27.513	**<2e-16**
Gb	–0.078	0.015	–5.061	**1.29e-06**
Gc	–0.107	0.015	–6.955	**1.23e-10**
Gd	–0.075	0.015	–4.879	**2.85e-06**
Ge	–0.130	0.015	–8.435	**3.65e-14**
Gf	–0.152	0.015	–9.862	**<2e-16**
**Approximate significance of smooth terms ** ***f(GxT)*** **:**	**edf**	**Ref.df**	**F**	***P***
s(T):Ga	3.86	3.89	319.5	**<2e-16**
s(T):Gb	3.87	3.89	314.3	**<2e-16**
s(T):Gc	3.89	3.89	299.7	**<2e-16**
s(T):Gd	3.88	3.89	250.4	**<2e-16**
s(T):Ge	3.87	3.89	234.2	**<2e-16**
s(T):Gf	3.81	3.89	216.5	**<2e-16**
s(T):Gg	3.77	3.88	304.7	**<2e-16**
GCV score = 0.0037034	n = 175	**R^2^_adj_ = 0.96**		

*d) % reduction = (T) + (G) + f(GxT) + ε*				
Parametric coefficients:	Estimate	Std. Error	t value	Pr(>|t|)
Intercept	106.56	2.55	41.733	**<2e-16**
T	–4.31	0.29	–19.777	**<2e-16**
Gb	–11.06	1.77	–6.240	**4.86e-09**
Gc	–17.49	1.77	–9.867	**<2e-16**
Gd	–15.49	1.77	–8.739	**6.45e-15**
Ge	–17.59	1.77	–9.925	**<2e-16**
Gf	–18.74	1.77	–10.575	**<2e-16**
Gg	–21.40	1.77	–12.075	**<2e-16**
**Approximate significance of smooth terms ** ***f(GxT)*** **:**	**edf**	**Ref.df**	**F**	***P***
s(T):Ga	3.84	3.89	182.3	**<2e-16**
s(T):Gb	3.85	3.89	263.3	**<2e-16**
s(T):Gc	3.88	3.89	297.0	**<2e-16**
s(T):Gd	3.88	3.89	227.0	**<2e-16**
s(T):Ge	3.85	3.89	181.7	**<2e-16**
s(T):Gf	3.86	3.89	173.4	**<2e-16**
s(T):Gg	3.84	3.89	447.7	**<2e-16**
GCV score = 49.002	n = 17	**R^2^_adj_ = 0.97**		

Bold faced values indicate p<0.01 significance.

The highest *P uninf* occurred at 21°C probably due to fast host population growth and complete loss of the parasite, while the lowest *P uninf* occurred at 16°C due to high infection related losses (Figures 3A (observed data) and 4A (GAM predictions)). Conversely, the highest *P inf* occurred at 16°C and the lowest at 21°C (Figs. 3B and 4B). In general, infected host cells carried single infections, multiple infections per host cell only occurred when infection prevalence was extremely high and the availability of uninfected hosts became limiting. Hence, the *P spor* showed also the highest production at 16°C and the lowest at 1°C and 21°C (data not shown). The formation of resting spores at 1°C resulted in a loss of prevalence (i.e. negative *P prev*) over time at 1°C, while the loss of prevalence at 21°C was caused by the death of the parasite population (Figs. 3C and 4C). The positive *P prev* at intermediate temperatures (11 and 16°C) suggested that parasite production rates surpassed those of the host. This was also reflected in the*% reduction* patterns (Figs. 3D and 4D). Impact of infection was highest at the intermediate temperatures (positive values in *% reduction of uninfected cells*) and lowest at the two temperature extremes. Negative values in *% reduction* indicated that the production of uninfected cells in the experimental units surpassed that in the controls.

To exclude that we confounded genotype effects with host cell bio-volume effects on host susceptibility and parasite productivity, we checked for the respective explanatory power of predictors a) host cell bio-volume and b) genotype using ANOVA models (see file S2 for methods and results). The model including genotype provided higher predictive power for host and parasite productivity measures, therefore all results were interpreted in the light of genotype effects.

## Discussion

### General temperature effects

Species-level host and parasite rate of change per day showed a typical left skewed, unimodal relationship across temperatures with maximal performance temperatures near their upper tolerance limits [43]. The net loss of infection prevalence at both temperature extremes showed that the thermal activity range of the tested parasite genotype was narrower than that of its host. However the mechanism at work was different for either temperature extreme. At 1°C the parasite was still able to reproduce but formed mostly resting spores which remain inactive as long as the conditions are adverse for the parasite. Here, the loss of prevalence was caused by the host population growth rate exceeding the parasite population growth rate so that the proportion of infected cells was constantly diluted by new, uninfected cells (Fig. 3C). Hence, the disease was present, but showed such slow dynamics that it was contained at very low levels in the host population. At 21°C, the loss of prevalence was caused by the parasite dying within a few days which freed the host population from parasite pressure as reflected in the low *% reduction* of exposed but uninfected host cells at this temperature (see Figs. 3D and 4D). Also at 21°C, the production of uninfected host cells in some experimental units surpassed that of controls which may be a result of increased nutrient recycling from (few) infected, dying cells or an indication of unexpected high variance in host carrying capacity at that temperature.

The narrower thermal activity range of the tested parasite genotype allowed the host two thermal refugia of low or no disease pressure. We tested only one parasite genotype, and given that parasite genetic diversity is likely also expressed in phenotypic diversity, the actual species-level reaction norm of the parasite may look slightly different. Nevertheless, the occurrence of the “cold” thermal refuge for the host has been described in earlier field studies [26] and in a laboratory study on a closely related parasite species, *Rhizophydium planktonicum* [36]. Similarly, thermal refugia have also been described in other species pairs such as *Daphnia magna* and its bacterial parasite *Pasteuriaramosa* where disease severity decreased drastically with temperature [11]. One of the most striking examples of thermal refugia is the induction of behavioural fever [44]. Amphibians are able to clear chytrid infections by seeking high temperature environments [45], [46]. Desert locusts use behavioural fever to control fungal infections to survive long enough to produce offspring [46]. Phytoplankton species such as the diatom *Asterionella* have, of course, limited capacity to actively choose their temperature environment but show a similar respite from fungal infection during cold winters and at the height of summer when surface water temperatures favour the host but not the parasite. Such thermal refugia may seem short-lived and of little consequence, but nevertheless have measurable impact, for instance in the *Asterionella* population dynamics in Lake Maarsseveen. The occurrence and timing of the “cold” refugium determines the occurrence and size of the *Asterionella* spring-bloom and therefore sets the stage for the seasonal phytoplankton succession and food-web dynamics in the lake [12]. The “hot” refugium” may facilitate the occurrence of high density summer/autumn blooms of *Asterionella*, as epidemics of the chytrid reach only low infection prevalence despite high host density due the parasitès lower thermal maximum [47]. Such summer blooms, in turn, are a poor food source for cladocerans as *Asterionella* is basically not ingestible for these zooplankters [48].

### Genotype and genotype-by-environment interactions

Our experiment also showed that host genotypes differed in their overall susceptibility to disease, indicating that they possess variation in disease resistance traits. Thermal variation in the environment, however, is likely to hinder any directional selection against susceptible genotypes as the susceptibility ranking order of the tested host genotypes varied significantly with temperature (Fig. 3A–D). Therefore, it is not possible to predict the strength and exact direction of parasite selective pressure on any given host genotype from one environment to another. The influence of the thermal environment on host genotype-specific susceptibility to disease has been shown in a number of invertebrate-parasite systems [6], [10], [11], [49] and in vascular plants [50]. Context dependency of the host genotype-specific response to infection (GxE interactions) may contribute to the observed high level of genetic diversity in natural *Asterionella* populations [31] under the pre-condition that different host genotypes vary in their susceptibility to infection under different environments (as found in this study). However, temperature is only one (although an important one) of the regulating factors in a complex environment. Changes in light and oxygen saturation with watercolumn depth, seasonal nutrient and pH variation or the presence of competitors and predators may all add their own twist to host-parasite interactions.

## Conclusions

Host and chytrid parasite thermal tolerance ranges do not necessarily overlap fully. If the thermal tolerance range of the parasite is narrower than that of its host, the host can benefit from thermal refugia of low or no disease pressure. This seems to be the case in chytrid-*Asterionella* system but also in the chytrid-amphibian systems. If changes in temperature patterns due to climate warming affect the duration and timing of such thermal refugia for the host, this may have important and potentially unexpected consequences for parasite and host population dynamics. Warming may stimulate the spread of disease by removing cold temperature refugia; although the loss of such host refugia may also result in the paradoxical subsequent loss of host blooms and parasite epidemics (see for example [12]). Hence, the outcome of climate warming on the spread and severity of diseases is not always straightforward to predict. Furthermore, the mechanisms underlying the occurrence of host refugia may vary from reduced parasite population growth to parasite dormancy to extinction of the parasites. Which of these processes are in operation may have implications for disease re-occurrence or re-invasion from resting stages and for host pre-adaptation to disease. Selection on the *Asterionella* genotypes can then be driven by different factors (environment or parasite), which may have consequences in the potential for host-parasite co-evolution. In any thermal refugium, the host population is freed of parasite mediated selection but experiences abiotic selection pressures. If host genotypes show different performance ranking orders under abiotic stress than under parasite pressure, then selection in the thermal refugia may also favour a different set of host genotypes, disrupt any directional selection for increased parasite resistance in the host population, and cause the host population to lose, to some extent, any pre-adaptation to the parasite. This would increase the infection success of the parasite when re-invading from resting stages. Such examples indicate that the mechanisms behind (temporary) disappearance of disease need to be taken into account in theoretical approaches as well as in the management of infectious diseases.

## Supporting Information

File S1 **Fingerprinting of** ***Asterionellaformosa*** **isolates**. Description of the AFLP fingerprinting and the statistical analysis methods used for the assessment of genetic diversity in the experimental *Asterionellaformosa* genotypes.
(DOCX)

File S2 **ANOVA models testing co-linearity between predictors host genotype and host cell-size**. To assess whether genotype or host cell-size is the more appropriate predictor for host and parasite productivity, two ANOVA models were compared including either temperature and host genotype (model 1, tables S2.1 and S2.4) or temperature and host cell-size (model 2, tables S2.2 and S2.5). Response variables were net production of uninfected cells (see S2.1 and S2.2) and net production of infected cells in parasite exposed cultures (see S2.4 and S2.5).
(DOCX)
