# Supplementary material for: Temperature Alters Host Genotype-Specific Susceptibility to Chytrid Infection
Source: PLoS One. 2013 Aug 26;8(8):e71737. doi: 10.1371/journal.pone.0071737 (PMC3753301; doi:10.1371/journal.pone.0071737)
Supplement: File S1 — Fingerprinting of Asterionellaformosa isolates. Description of the AFLP fingerprinting and the statistical analysis methods used for the assessment of genetic diversity in the experimental Asterionellaformosa genotypes. (DOCX) [file pone.0071737.s001.docx]

**S1 Fingerprinting of *Asterionella formosa* isolates**

*Genetic analysis*

To assess the genetic diversity among *Asterionella formosa* genotypes they were ﬁngerprinted by AFLP markers. AFLPs are sensitive and reproducible markers that can reveal genotypic and phylogenetic relationships [1] and provide useful information on population genetic structure where other markers lack sufﬁcient resolution. For instance, AFLP markers have been used to characterize the clonal structure of a single algal bloom sample [2]. *Asterionella* DNA was extracted with a Qiagen DNeasy Plant Mini Kit (Qiagen N.V., Venlo, the Netherlands) with a modiﬁcation of the lysis step. A volume of 50 mL of dense *Asterionella* cultures was centrifuged at 740g for 10 min. The supernatant was discarded and the remaining pellet was transferred into a fresh tube together with 0.5 g of 0.1 mm zirconium beads and 300 µLlysis buffer. The lysis buffer consisted of 10 mM Tris 1 M, 1 mM EDTA pH8 and 0.5% SDS. Then the sample was bead-beaten three times with a mini bead beater (Biospec Products, Bartlesville, OK, USA) at 840g for 30 s per round and cooled on ice between each round. After adding 100 µg/mL Proteinase K, the sample was vortexed, and then incubated at 60 ° C for 1 h. Thereafter it was further processed according to the Qiagen DNeasy Plant Mini Kit protocol from the ﬁrst precipitation step onward. The AFLP analysis was performed by Keygene ® (Keygene N.V., Wageningen, The Netherlands) using the same 4 EcoRI/Msel AFLP primer combinations as reported in De Bruin et al. [3], (i) Eco + GA & Mse + AT, (ii) Eco + GA & Mse + CC, (iii) Eco + GA & Mse + CG, (iv) Eco + GC & Mse + AC.

*Statistical analysis*

For the AFLP analysis, each locus was treated as a separate character and scored for presence or absence, yielding a binary data matrix. This matrix was used to estimate genetic similarity of all possible pair-wise combinations of the tested genotypes using Jaccard’s similarity coefﬁcient [4]. Jaccard’s similarity ignores shared absences and the coefﬁcient ranges from zero (no similarity) to one (complete similarity; [5]). Cluster analysis using the unweighted pair-group method using arithmetic means procedure (UPGMA) was performed to generate a dendrogram visualizing the similarity of the tested genotypes. The cophenetic correlation coefﬁcient was calculated between the cophenetic matrix and the Jaccard’s similarity matrix to evaluate to what extent the similarity matrix supported the dendrogram [6]. Resampling the dataset showed support for most nodes, except for the node distinguishing S122 and S43. Nevertheless, given the empirical data, this dendrogram is the best possible representation of the data.

**References**

1. Rademaker JLW, Hoste B, Louws FJ, Kersters K, Swings J, et al. (2000) Comparison of AFLP and rep-PCR genomic fingerprinting with DNA-DNA homology studies: *Xanthomonas* as a model system. Int J Syst Evol Microbiol 50: 665-677.

2. John U, Groben R, Beszteri B, Medlin L (2004) Utility of Amplified Fragment Length Polymorphisms (AFLP) to Analyse Genetic Structures within the *Alexandrium tamarense* Species Complex. Protist 155: 169-179.

3. De Bruin A, Ibelings BW, Rijkeboer M, Brehm M, van Donk E (2004) Genetic variation in *Asterionella formosa* (Bacillariophyceae): Is it linked to frequent epidemics of host-specific parasitic fungi? J Phycol 40: 823-830.

4. Jaccard P (1901) Etude comparative de la distribution florale dans une portion des Alpes et du Jura. Bull. Soc. vaud. sci. nat. 37: 547-579.

5. Bonin A, Ehrich D, Manel S (2007) Statistical analysis of amplified fragment length polymorphism data: a toolbox for molecular ecologists and evolutionists. Mol Ecol 16: 3737-3758.

6. Rohlf FJ, Sokal RR (1981) Comparing numerical taxonomic studies. Syst Zool 30: 459-490.
