# Supplementary material for: Temperature Alters Host Genotype-Specific Susceptibility to Chytrid Infection
Source: PLoS One. 2013 Aug 26;8(8):e71737. doi: 10.1371/journal.pone.0071737 (PMC3753301; doi:10.1371/journal.pone.0071737)
Supplement: File S2 — ANOVA models testing co-linearity between predictors host genotype and host cell-size. To assess whether genotype or host cell-size is the more appropriate predictor for host and parasite productivity, two ANOVA models were compared including either temperature and host genotype (model 1, tables S2.1 and S2.4) or temperature and host cell-size (model 2, tables S2.2 and S2.5). Response variables were net production of uninfected cells (see S2.1 and S2.2) and net production of infected cells in parasite exposed cultures (see S2.4 and S2.5). (DOCX) [file pone.0071737.s002.docx]

**S2 ANOVA models testing co-linearity between predictors host genotype and host cell-size**

We tested whether host genotype or host cell-size is the more appropriate predictor for host and parasite productivity by comparing two ANOVA models including either temperature and host genotype (model 1, tables S2.1 and S2.4) or temperature and host cell-size (model 2, tables S2.2 and S2.5). Response variables were net production of uninfected cells and net production of infected cells in parasite exposed cultures.

*Size measurements*

To assess host cell-volume of uninfected *Asterionella*, length and width measurements of 30 uninfected cells for each experimental unit in the temperature treatments 6 °C and 16 °C were used to calculate cell-volume, assuming that cells resemble an elongated cuboid. For all measurements (in µm) an inverted microscope and the image analysis program Cell-D (Olympus Soft Imaging System, Münster, Germany) were used.

*Statistical Analysis*

The extent of co-linearity between predictors host genotype and host cell-size on host and parasite productivity was tested with two ANOVA models comparing the predictive power of temperature and genotype (model one) or temperature and *luninf* cell volume (model two). The models were tested for fit and the whole model R^2^_adj_ was used as decision criterion to assess which model, i.e. which predictor (host genotype or size), had more predictive power. All statistical analyses and plot graphing were carried out in R [[1](#_ENREF_1)] and SigmaPlot 11.0 (Systat Software, San Jose, U.S.).

*Results*

For both response variables (net production of uninfected cells and net production of infected cells in exposed cultures) there was a significant difference between model fits (tables S2.3 and S2.6). In both cases the model including host genotype had higher predictive power than the model including host cell-size. In light of these results, the analysis of the experiment (presented in the main document) proceeded with temperature and genotype as predictors.

**Table S2.1**: results of the factorial ANOVA on the effect of temperature (T) and host genotype (Ga-g) and their interaction (T:Ga-g) on the net production of uninfected cells in parasite exposed cultures in the temperature treatments 6 and 16°C.

| ***P uninf ~ (T) + (G) + (TxG)*** | Estimate | Std. Error | t value | Pr(>\|t\|) |
| --- | --- | --- | --- | --- |
| (Intercept) | 134776.1 | 7047.4 | 19.12 | **< 2e-16** |
| T | -8252.9 | 583.2 | -14.15 | **< 2e-16** |
| Gb | 50944.2 | 9966.5 | 5.11 | **7.41e-06** |
| Gc | -24552.2 | 9966.5 | -2.46 | **0.018** |
| Gd | 26190.1 | 9966.5 | 2.63 | **0.012** |
| Ge | -29939.5 | 9966.5 | -3.00 | **0.004** |
| Gf | -79704.4 | 9966.5 | -7.99 | **5.65e-10** |
| Gg | -73431.5 | 9966.5 | -7.37 | **4.35e-09** |
| T:Gb | -2933.5 | 824.8 | -3.56 | **0.001** |
| T:Gc | 2309.2 | 824.8 | 2.80 | **0.008** |
| T:Gd | -1235.5 | 824.8 | -1.49 | 0.142 |
| T:Ge | 4442.6 | 824.8 | 5.39 | **3.02e-06** |
| T:Gf | 9209.6 | 824.8 | 11.17 | **3.78e-14** |
| T:Gg | 7049.3 | 824.8 | 8.55 | **9.80e-11** |

Residual standard error: 8248 on 42 degrees of freedom

Multiple R^2^: 0.9614, **R^2^_adj_: 0.9494**, F_(13,42)_ = 80.46, *P* < 2.2e-16

**Table S2.2:** results of the factorial ANOVA on the effect of temperature (T), host cell-size (Size) and their interaction (T:Size) on the production of uninfected cells in parasite exposed cultures in the temperature treatments 6 and 16°C.

| ***P uninf ~ (T) + (Size) + (TxSize)*** | Estimate | Std. Error | t value | Pr(>\|t\|) |
| --- | --- | --- | --- | --- |
| (Intercept) | 271391.1 | 71706.5 | 3.79 | **0.001** |
| T | -23025.4 | 5927.1 | -3.89 | **0.001** |
| Size | -4359.8 | 1811.2 | -2.41 | **0.019** |
| T:Size | 522.4 | 168.1 | 3.11 | **0.003** |

Residual standard error: 22200 on 52 degrees of freedom

Multiple R^2^: 0.6539, **R^2^_adj_: 0.6339**, F_(3,52)_ = 32.75, *P* = 4.988e-12

**Table S2.3:** result of the comparison of models S2.1 and S 2.2

Model 1: *P uninf ~ (T) + (G) + (TxG)*

Model 2: *P uninf* ~ (T) + (Size) + (TxSize)

|  | Res.Df | RSS | Df | Sum of Sq | F | Pr(>F) |
| --- | --- | --- | --- | --- | --- | --- |
| 1 | 42 | 2.86e+09 |  |  |  |  |
| 2 | 52 | 2.56e+10 | -10 | -2.2762e+10 | 33.457 | **< 2.2e-16** |

**Table S2.4:** results of the factorial ANOVA on the effect of temperature and host genotype on the net production of infected cells in parasite exposed cultures in the temperature treatments 6 and 16°C.

| ***Sqrt P inf ~ (T) + (G) + (TxG)*** | Estimate | Std. Error | t value | Pr(>\|t\|) |
| --- | --- | --- | --- | --- |
| (Intercept) | 237.3 | 11.05 | 21.48 | **< 2e-16** |
| T | 3.7 | 0.91 | 4.05 | **0.001** |
| Gb | -123.0 | 15.6 | -7.87 | **8.44e-10** |
| Gc | -178.5 | 15.6 | -11.42 | **1.83e-14** |
| Gd | -124.6 | 15.6 | -7.98 | **6.07e-10** |
| Ge | -246.9 | 15.6 | -15.79 | **< 2e-16** |
| Gf | -253.1 | 15.6 | -16.19 | **< 2e-16** |
| Gg | -205.4 | 15.6 | -13.15 | **< 2e-16** |
| T:Gb | 10.3 | 1.29 | 7.93 | **7.08e-10** |
| T:Gc | 17.3 | 1.29 | 13.39 | **< 2e-16** |
| T:Gd | 9.6 | 1.29 | 7.42 | **3.62e-09** |
| T:G | 19.9 | 1.29 | 15.45 | **< 2e-16** |
| T:Gf | 19.1 | 1.29 | 14.77 | **< 2e-16** |
| T:Gg | 16.2 | 1.2931 | 12.49 | **9.90e-16** |

Residual standard error: 12.93 on 42 degrees of freedom

Multiple R^2^: 0.9855, **R^2^_adj_: 0.981**, F_(13,42)_ = 219.4, *P* < 2.2e-16

**Table S2.5:** results of the factorial ANOVA on the effect of temperature and host cell-size on the net production of infected cells in parasite exposed cultures in the temperature treatments 6 and 16°C.

| ***Sqrt P inf ~ (T) + (Size)+ (TxSize)*** | Estimate | Std. Error | t value | Pr(>\|t\|) |
| --- | --- | --- | --- | --- |
| (Intercept) | -21.4 | 128.82 | -0.17 | 0.869 |
| T | 26.7 | 10.65 | 2.51 | **0.015** |
| Size | 2.6 | 3.25 | 0.81 | 0.421 |
| T:Size | -0.3 | 0.30 | -0.95 | 0.349 |

Residual standard error: 39.88 on 52 degrees of freedom

Multiple R^2^: 0.8291, **R^2^_adj_: 0.8193**, F_(3,52)_ = 84.12, *P* < 2.2e-16

**Table S2.6:** results of the comparison of models S2.4 and S2.5

Model 1: *Sqrt P inf ~ (T) + (G) + (TxG)*

Model 2: *Sqrt P inf ~ (T) + (Size) + (TxSize)*

|  | Res.Df | RSS | Df | Sum of Sq | F | Pr(>F) |
| --- | --- | --- | --- | --- | --- | --- |
| 1 | 42 | 7023 |  |  |  |  |
| 2 | 52 | 82690 | -10 | -75668 | 45.26 | **< 2.2e-16** |

**References**

1. R Development Core Team (2011) R: A language and environment for statistical computing Vienna, Austria: R Foundation for Statistical Computing.
